# Proton therapy for isolated local regional recurrence of breast cancer after mastectomy alone

**DOI:** 10.3389/fonc.2022.925078

**Published:** 2022-11-28

**Authors:** Brady S. Laughlin, Ronik S. Bhangoo, Joshua R. Niska, Cameron S. Thorpe, Marlene E. Girardo, Justin D. Anderson, Heidi E. Kosiorek, Lisa A. McGee, William F. Hartsell, John H. Chang, Carl J. Rossi, Henry K. Tsai, Isabelle J. Choi, Carlos E. Vargas

**Affiliations:** ^1^ Department of Radiation Oncology Mayo Clinic, Phoenix, AZ, United States; ^2^ Health Sciences Research, Division of Biostatistics, Mayo Clinic, Scottsdale, AZ, United States; ^3^ Northwestern Medicine Chicago Proton Center, Warrenville, IL, United States; ^4^ ProCure Proton Therapy Center, Oklahoma City, OK, United States; ^5^ Scripps Proton Therapy Center, San Diego, CA, United States; ^6^ ProCure Proton Therapy Center, Somerset, NJ, United States; ^7^ New York Proton Center, New York, NY, United States

**Keywords:** breast cancer, post-mastectomy radiotherapy, protons, local recurrence, PMRT

## Abstract

**Purpose/Objectives:**

To assess adverse events (AEs) and disease-specific outcomes after proton therapy for isolated local-regional recurrence (LRR) of breast cancer after mastectomy without prior radiotherapy (RT).

**Materials/Methods:**

Patients were identified from a multi-institutional prospective registry and included if diagnosed with invasive breast cancer, initially underwent mastectomy without adjuvant RT, experienced an LRR, and subsequently underwent salvage treatment, including proton therapy. Follow-up and cancer outcomes were measured from the date of RT completion.

**Results:**

Nineteen patients were included. Seventeen patients were treated with proton therapy to the chest wall and comprehensive regional lymphatics (17/19, 90%). Maximum grade AE was grade 2 in 13 (69%) patients and grade 3 in 4 (21%) patients. All patients with grade 3 AE received > 60 GyE (p=0.04, Spearman correlation coefficient=0.5). At the last follow-up, 90% of patients were alive with no LRR or distant recurrence.

**Conclusions:**

For breast cancer patients with isolated LRR after initial mastectomy without adjuvant RT, proton therapy is well-tolerated in the salvage setting with excellent loco-regional control. All grade 3 AEs occurred in patients receiving > 60 GyE.

## Introduction

In the United States, post-mastectomy radiotherapy (PMRT) is generally recommended for patients with node-positive or T3-4 breast cancers (1, 2). Historically, loco-regional recurrence (LRR) rates after mastectomy without PMRT were about 20-30%. However, for T1-2 tumors treated with modern chemotherapy, LRR rates are closer to 10%. Salvage therapy for LRR after mastectomy typically consists of a multimodality approach, including surgery, radiotherapy, and systemic therapy (3). Loco-regional control (LRC) after salvage treatment with radiotherapy for a first LRR after mastectomy has been estimated to be 60-70%, with patients who progress also experiencing distant metastases (4, 5).

Although target delineation is not standardized for salvage radiotherapy, one older study did show that treating the chest wall (CW) and supraclavicular fossa resulted in better LRC at 5 and 10 years compared to the CW alone (5-year LRC 75% vs. 36%; 10-year LRC 63% vs. 18%) (5). The challenges of maintaining target coverage while minimizing dose to underlying heart and lungs are particularly evident when attempting to treat the internal mammary lymph node chains (IMNs), left-sided breast tumors, CW after immediate reconstruction, and otherwise unfavorable CW anatomy. Total nodal irradiation may be preferable in this setting, as additional curative therapy may not be possible after a second LRR.

The use of proton therapy is an attractive option for patients who have experienced an isolated LRR after initial mastectomy without adjuvant RT. Protons exhibit minimal exit dose, as they deposit their dose at the end of their range, known as the Bragg peak (6). Treatment planning studies have demonstrated significantly decreased heart and lung doses with proton than photon PMRT (7, 8). To date, published clinical proton PMRT series have focused on the adjuvant or re-irradiation settings (9–11).

From a prospectively maintained multi-institutional registry, we report an initial clinical experience with proton therapy for isolated LRR in patients with breast cancer initially treated with mastectomy without adjuvant RT.

## Methods

### Study design and patient selection

All patients signed consent to participate in an IRB-approved multi-institutional prospective registry of patients treated with proton therapy in the United States (Proton Collaborative Group, PCG REG001-09). Patients were included in this study if they were diagnosed with invasive breast cancer, initially underwent mastectomy without adjuvant RT, experienced an isolated LRR, and subsequently underwent salvage treatment, including proton therapy. We identified patients who began proton therapy between 2013 and 2016. Relevant patient and tumor characteristics were collected using the electronic medical record.

Components of salvage treatment may have included systemic therapy, surgery, and proton beam radiation. Systemic therapy (including hormone therapy or chemotherapy) could have been administered in the initial treatment course following recurrence. Patients included in this analysis were not required to have undergone salvage surgery prior to proton therapy. Patients were excluded if they had metastatic disease at the time of proton therapy.

### Radiotherapy

The treating physician and subject decided on proton therapy with insurance approval. RT dose, treatment technique, target delineation, and organ at risk dose constraints were also at the treating physician’s discretion, planning team, and institutional guidelines. Passive scatter, uniform scanning, and pencil beam scanning proton treatment were allowed.

All patients received RT to the chest wall. The treating physician determined the inclusion of nodal irradiation. Regional nodal irradiation (RNI) is radiation to the axillary, supraclavicular, and IMN lymph node chains.

### Outcomes and adverse events

Follow-up and cancer outcomes were measured from the date of proton therapy completion. LRR was defined as CW or lymph node recurrence within the axillary, infraclavicular, supraclavicular, and/or internal mammary lymph node regions. Distant recurrence was defined as disease recurrence outside of the regions specified above.

Adverse events (AEs) were graded according to the Common Terminology Criteria for Adverse Events (CTCAE) version 4.0. Acute AEs occurred within six months of the start date, whereas late AEs occurred or persisted beyond 6 months from the start date.

### Statistical analysis

Clinical and treatment characteristics were tested for association with AE grade using Fisher’s exact test for categorical and Kruskal-Wallis test for continuous variables. Spearman correlation coefficients were calculated to assess the strength of associations using criteria published by Cohen: low correlation, 0.10 to 0.29; moderate correlation, 0.30 to 0.49; high correlation, > 0.50 (12). Univariate Cox models were used to determine variables associated with grade 3 adverse events. Kaplan Meier curves were used to determine clinical outcome rates. Statistical analysis was performed using SAS v 9.4 (SAS Institute Inc.). P values were derived from two-tailed tests. P values less than 0.05 were considered statistically significant.

## Results

### Patient and tumor characteristics

Nineteen patients were included. **Table 1** presents patient and tumor characteristics. The median follow-up from completion of proton therapy was 13.4 months. At first recurrence, the most common T stage was cT1 (8/19 patients, 42%). At first isolated LRR, most patients were cN0 (13/19, 68%). Most patients had grade 3 (10/19, 53%) ductal carcinoma (15/19, 79%), estrogen receptor-positive (12/19, 63%), progesterone receptor positive (12/19, 63%), and human epidermal growth factor receptor 2 (HER-2) negative (15/19, 79%). Five patients had triple negative breast cancer (5/19, 26%).

**Table 1:** Patient and treatment characteristics (N = 19).

**Age at Proton RT** [Years, Median (Range)]	48.7 (32.4-84.4)
**Follow-up** [Months, Median (Range)]	13.4 (0.0-36.6)
**Disease Laterality** [L/R]	11 (57.9%)/8 (42.1%)
**Initial Surgery**	
Mast	2 (10.5%)
Mast+ALND	8 (42.1%)
Mast+NOS	1 (5.3%)
Mast+SLNB	8 (42.1%)
**T Stage at Recurrence**	
cT1	8 (42.1%)
cT2	4 (21.1%)
cT4b	3 (15.8%)
cTx	4 (21.1%)
**N Stage at Recurrence**	
cN0	13 (68.4%)
cN1	1 (5.3%)
cN2b	2 (15.8%)
cN3a	1 (5.3%)
cN3c	1 (5.3%)
**Molecular Subtype of Recurrence**	
ER/PR+, HER2 normal, grade 1-2	7 (36.8%)
Triple Negative	5 (26.3%)
ER/PR+, HER2 normal, grade 3	3 (15.8%)
Triple Positive	2 (10.5%)
HER2+	1 (5.3%)
NR	1 (5.3%)
**Salvage Surgery**	
ALND	1 (5.3%)
Bx (i.e. no surgery)	4 (21.1%)
WLE	12 (63.2%)
WLE+ALND	1 (5.3%)
WLE+SLNB	1 (5.3%)
**Margin Status of Salvage Surgery**	
R0	11 (57.9%)
R1	2 (10.5%)
R2	2 (10.5%)
Bx Only	4 (21.1%)
**Proton Technology**	
NR	2 (10.5%)
Uniform Scanning	16 (84.2%)
PBS	1 (5.3%)
**Proton PTV**	
CW	1 (5.3%)
CW+IMN	1 (5.3%)
CW+RNI	17 (89.5%)
**Proton Fractions** [Median (Range)]	33.0 (26.0-38.0)
**Proton Dose** [Median (Range), GyE]	60.5 (47.2-70.4)

*RT, radiotherapy; Mast, mastectomy; NOS, not otherwise specified; SLNB, sentinel lymph node biopsy; NR, not reported; ALND, axillary lymph node dissection; Bx, biopsy; WLE, wide local exicision; PBS, pencil beam scanning; CW, chest wall; IMN, internal mammary lymph nodes; RNI, regional nodal irradiation.

### Salvage treatment characteristics

Salvage treatment characteristics are located in **Table 1**. Most patients (12/19, 63%) underwent wide local excision only. Of the patients who underwent salvage surgery, only 73% (11/15) achieved R0 resection. Four (21%) patients underwent biopsy only. Adjuvant endocrine therapy was started in 9 (47%). Chemotherapy was delivered in 9 (47%) patients (2 (11%) neoadjuvant, 6 (32%) adjuvant, and 1 (5%)both neoadjuvant and adjuvant).

Most patients received proton therapy by uniform scanning technology (16/19, 84%). Seventeen patients were treated with proton therapy to the chest wall (CW) and regional nodes (17/19, 90%). One patient was treated to the CW alone (1/19, 5%), and another patient was treated to the CW and IMNs (1/19, 5%). The median proton therapy dose was 60.4 GyE (47.2-70.4) in a median of 33 (26–38) fractions.

### Adverse events

**Figure 1:**
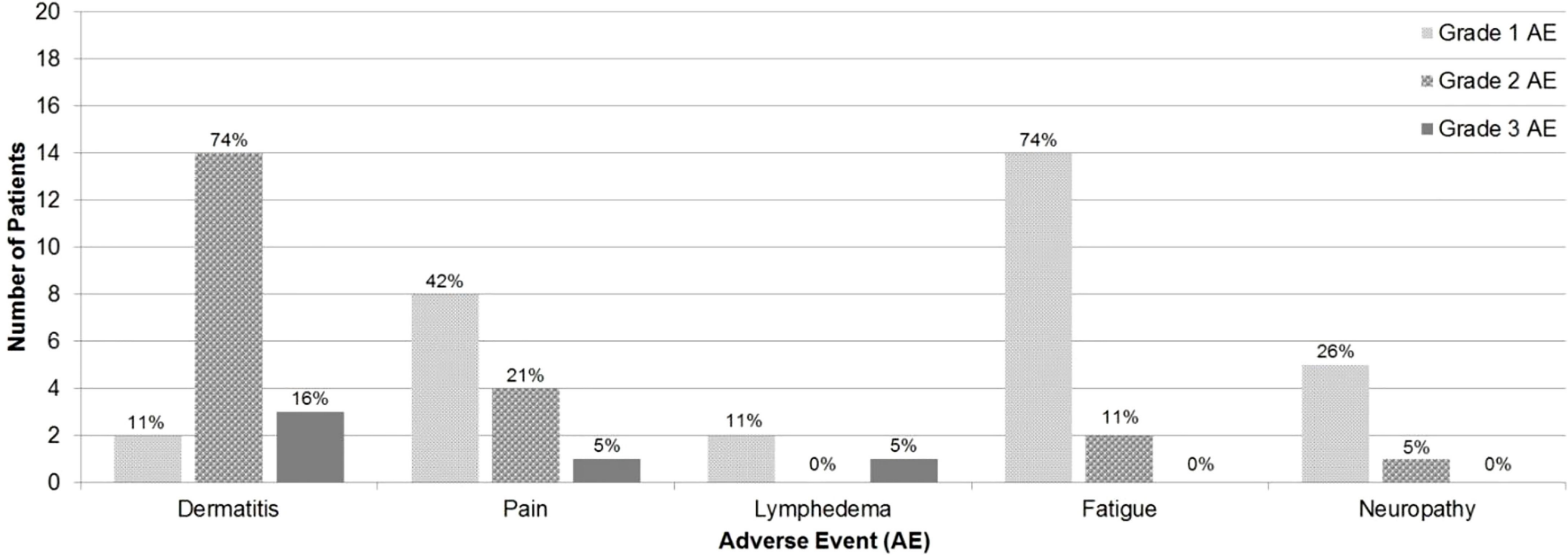
Maximum grade of adverse events by type.

Maximum grade AE was grade 2 in 13 (69%) patients and grade 3 in 4 (21%) patients. No patients experienced grade 4 or grade 5 AEs. Acute AEs were as follows: dermatitis (63% grade 2, 11% grade 3), pain (21% grade 2, 5% grade 3), fatigue (11% grade 2), and neuropathy (5% grade 2). Late AEs were as follows: dermatitis (11% grade 2, 5% grade 3) and lymphedema (5% grade 3) (**Figure 1**).

Univariate analysis was performed for maximum grade ≥3 AEs according to the following clinical and treatment variables: smoking status (p=1.0), BMI > 25 (p=0.09), median age during proton therapy (p=0.92), median time interval to isolated LRR (p=0.69), chemotherapy at initial diagnosis or LRR (p=0.60), axillary lymph node dissection at initial diagnosis or LRR (p=0.58), IMN or T4b disease at LRR (p=0.07), proton therapy to gross disease (p=0.56), and proton therapy to CW and RNI (p=0.39).

**Figure 2:**
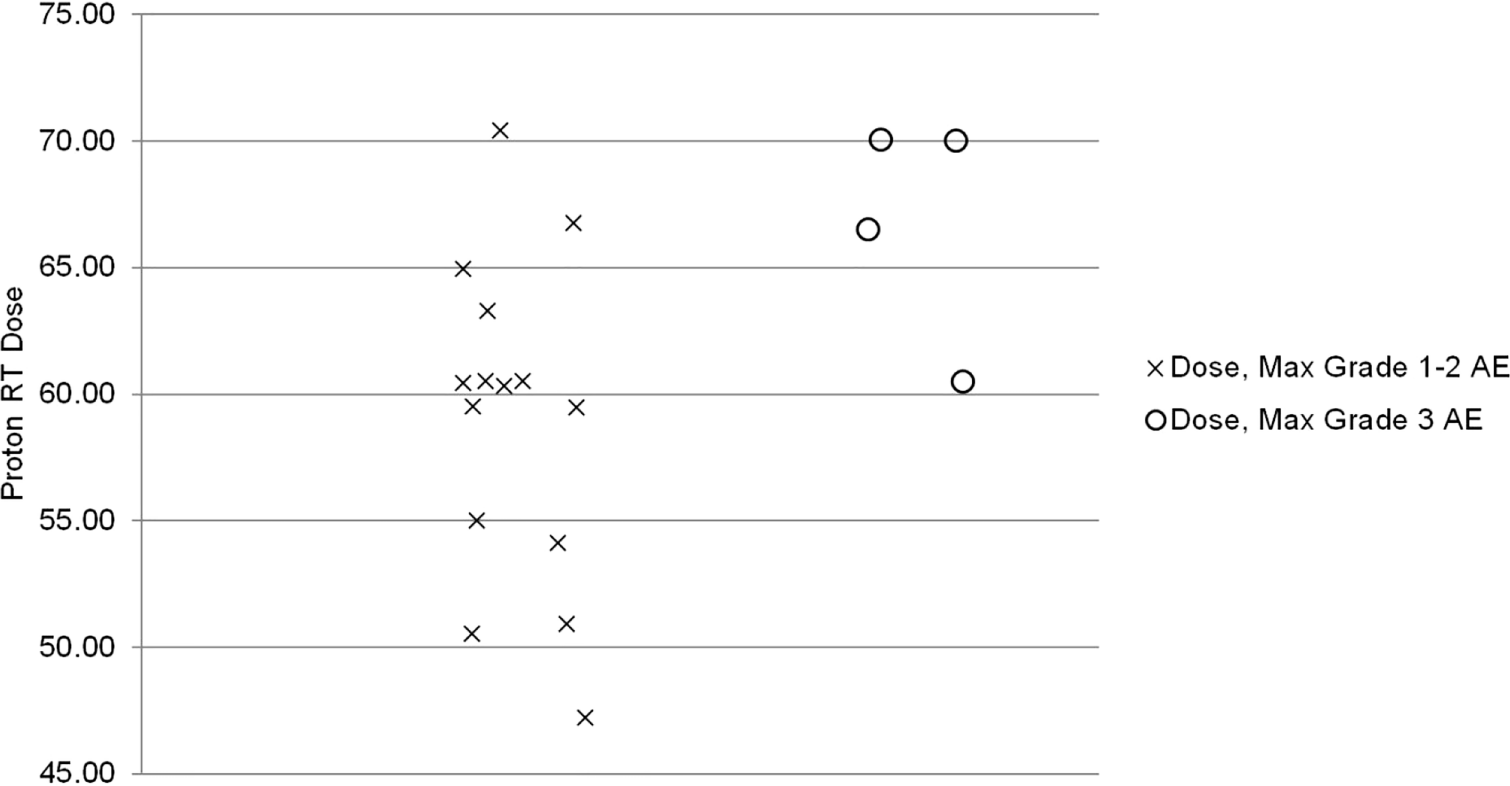
Proton therapy dose by maximum grade ≥3 adverse event (p = 0.04, rho = 0.50).

The median proton therapy dose was significantly associated with a maximum grade ≥3 AEs (p=0.04). All grade 3 AEs occurred in patients receiving > 60 GyE. The Spearman correlation coefficient (ρ) for the strength of association was 0.5 (high correlation p=0.04, **Figure 2**) (12).

### Outcomes

At the last follow-up, 17 (90%) patients were alive with no LRR or distant recurrence. One (5%) patient was alive with metastatic disease, and 1 (5%) patient had died of metastatic disease.

## Discussion

Loco-regional recurrence following mastectomy for breast cancer is not uncommon. In large prospective randomized trials, local recurrence rates following mastectomy range from 2 to 19% over long follow-up periods (6-19 years) (13–16). Proton therapy can be utilized for breast cancer patients who develop a loco-regional recurrence after initial mastectomy without adjuvant RT, providing excellent short-term LRC without increased risk of severe toxicity. After salvage proton therapy, LRC was 100% at the time of the last follow-up, with only 4 (21%) patients experiencing a grade ≥3 AE.

Published literature on proton therapy after mastectomy is primarily limited to the adjuvant setting, focusing on early toxicities (10, 17, 18). For 30 patients receiving PMRT with protons, Cuaron et al. reported rates of grade 2 dermatitis of 71.4% and grade 3 reconstructive complication in 3% (10). MacDonald and colleagues reported that 9 of 12 (75%) had grade 2 radiation dermatitis during their treatment (18). Similarly, the current study reports grade 2 and 3 dermatitis rates of 63% and 11%, respectively. Additionally, similar to the adjuvant setting in which total proton PMRT dose was associated with increased risk of grade ≥ 2 dermatitis, our study found a significant association between total proton therapy dose and maximum grade ≥ 3 AE (17). In our series, all patients who experienced a maximum grade ≥ 3 AE received a proton therapy dose > 60 GyE with a Spearman correlation coefficient of 0.5 (p=0.04). Thus, the proton therapy dose should be carefully considered to maximize LRC but minimize acute and long-term toxicities. Hypofractionation may be one strategy to decrease the risk of skin toxicity as a phase 3 randomized trial demonstrated that hypofractionation was associated with a significantly decreased incidence of grade 3 acute skin toxicity (3 vs. 8%, p<0.01) compared to standard fractionation (19).

Proton beam therapy as the treatment in the post-mastectomy setting has dosimetric and radiobiologic benefits, with increased sparing of heart and lung and reduced secondary malignancy risk. In a population-based case-control study of 2168 women who received radiotherapy for breast cancer, Darby et al. reported that rates of major coronary events increased by 7.4% per gray delivered to the heart (20). Verma and colleagues completed a comprehensive review of proton beam therapy in a PMRT setting and demonstrated that PBT may limit coronary events with mean heart doses to ¾ 1 Gy (21). Radiation pneumonitis is also extremely rare, with development occurring in 1 of 102 patients in four studies (10, 18, 21–23). The development of secondary malignancy has been estimated to have a standardized incidence ratio of 1.23 compared to 1.08 for patients not undergoing irradiation (24). Raptis and colleagues reported a reduction in secondary malignancy risk in the lungs from 0.31% with photons to 0.12% with protons (25). Risk of secondary malignancy in the contralateral breast was 0.1% with photons and negligible for protons (25). Overall, secondary malignancy was four times lower with proton therapy than with photons (0.15 vs. 0.6%) (25).

Breast reconstruction, as well as its complications, are important aspects for consideration for protons with PMRT. From a technical perspective, protons may improve target coverage and spare underlying lung and heart. In a dosimetric study comparing intensity modulated proton therapy (IMPT), 3D conformal photon/electron, and wide tangent plans for patients with bilateral breast implants receiving PMRT, there was similar target coverage with enhanced homogeneity with reduction of lung and heart dose (26). In a retrospective review of 52 total patients with 42 undergoing bilateral reconstruction with unilateral IMPT, Smith et al. reported a higher risk of surgical infection(HR 13.19, 95% CI 1.67–104.03, *p* = 0.0012) and unplanned surgical intervention (HR 9.86, 95% CI 1.24–78.67, *p* = 0.0068) (9). Additionally, reconstruction failure was much more common in re-irradiated breasts (8/51 (15.6%) vs. 2/42 (4.8%)) (9). Hypofractionation was also associated with higher rates of reconstruction complications (HR 4.73, 95% CI 1.39–16.11, *p* = 0.01) (9). The rates of reconstruction failure with protons are similar to rates reported for photon therapy. Fowble and colleagues reported an 18% reconstruction failure rate with photons, primarily attributable to infection (9, 27). Similarly, a systematic review of implant-related complications reported 20% reconstruction failure following irradiation after reconstruction (28).

This study has several limitations. The study is limited by its small cohort. However, given the relatively uncommon occurrence of isolated LRR after mastectomy alone and the limited availability of proton therapy to patients, this is a reasonable initial report on the use of proton therapy in this setting. Additionally, the role of salvage surgery was not standardized for all patients with isolated LRR. This could be attributable to the multi-institutional nature of this study and the different practice standards at each institution. However, this report presents the feasibility of proton therapy in the salvage setting for patients undergoing either biopsy or wide local excision with or without axillary lymph node dissection. Another limitation of our study is the lack of dosimetric analysis and correlation with toxicity.

This experience details the use of proton therapy specifically for isolated LRR after mastectomy alone. Grade 3 acute toxicity was correlated with proton therapy dose greater than 60 GyE. Analyses of larger cohorts with longer follow-up is needed to assess outcomes and long-term toxicity of patients undergoing proton therapy following LRR after mastectomy without adjuvant RT.

## Data availability statement

The raw data supporting the conclusions of this article will be made available by the authors, without undue reservation.

## Ethics statement

The studies involving human participants were reviewed and approved by Mayo Clinic Institutional Review Board. Written informed consent for participation was not required for this study in accordance with the national legislation and the institutional requirements.

## Author contributions

CV conceptualized the project. BL, RB, and JN wrote the manuscript. All authors contributed to the article and approved the submitted version.

## Conflict of interest

The authors declare that the research was conducted in the absence of any commercial or financial relationships that could be construed as a potential conflict of interest.

## Publisher’s note

All claims expressed in this article are solely those of the authors and do not necessarily represent those of their affiliated organizations, or those of the publisher, the editors and the reviewers. Any product that may be evaluated in this article, or claim that may be made by its manufacturer, is not guaranteed or endorsed by the publisher.
